# Supplementary material for: Chromothripsis during telomere crisis is independent of NHEJ, and consistent with a replicative origin
Source: Genome Res. 2019 May;29(5):737–49. doi: 10.1101/gr.240705.118 (PMC6499312; doi:10.1101/gr.240705.118)
Supplement: Supplemental Material [file supp_gr.240705.118_Supplemental_file_1.zip › contigs/annotated_contigs/DB53/contig.2.DB53_length_778_mean_cov_12.2197943445.docx]

**DB53_length_778_mean_cov_12.2197943445**

```
ATAACAAACATGAAAAAAAGAAAAAGCAAGGACCCTGGAAACTCCATACAATACTCTATTTTTTCCACTTTGGATACTTAACCTATCTC
 >chr8:88670546-88670952 + E=2e-223
TTTTTGGTAACTAAGATATGCTCTCAATAGCCAGAGCATAACAGAGAGAATCCATTTCACTCCAATGCTTATGATTATGACTAAGATTA

TAAGTTATATGCTTTTAAGATTGCCTTCATTCATGCAGTGTATAGTGAGAATTTAATTTGTAGCAAATATCTAACAGTGGATGGGAAGA

AAAATGGCTTTACACACAAGAGGTTGCATGCTATAATAAAAAGAATCATAGAGTATGAAGCAAGGCCAAACTAAATTTGAATCTTTGAC

TTCCAGGTTGGAAATATATAAAGTTTTAGCTGAGAAACCTTTAATGAG|TC|TCCATAGGGAGTCATTTCCAACCCAACCAGTCAGCAC
                                                 >chr8:88051669-88052043 - E=4e-210
TCCAGTTCACTGGCCCCTATCCAACAAATTGTCCTTAAAAATGTCAATCCCCAAATTCTCGAGGAGACTAATTTGAGTAATTACAAAAC

TCTGGTCTCCAGCACAGCCAGCCCTGCATGAATAACTCATTCTCTATTGCAATTCCCCTATCTCAATAAATTGGCTCTATCTAGGCAGC

TGGCAAGGTGAACCTGTTGGGTGGCTACACATACATTTTTTTTAACAAAAGAAACTGGAAGTGAGAAATAAAACCAAAAAGCAGTTTCT

TAGAAAGGGACAATAAAATAGATAAACCTCTAGTAAATCCCACCCCCCCAAAAAACCATGGGGAAATA
```
